# Cell Therapy of Congenital Corneal Diseases with Umbilical Mesenchymal Stem Cells: Lumican Null Mice

**DOI:** 10.1371/journal.pone.0010707

**Published:** 2010-05-19

**Authors:** Hongshan Liu, Jianhua Zhang, Chia-Yang Liu, I-Jong Wang, Martin Sieber, John Chang, James V. Jester, Winston W. Y. Kao

**Affiliations:** 1 Department of Ophthalmology, University of Cincinnati, Cincinnati, Ohio, United States of America; 2 Department of Ophthalmology, College of Medicine, National Taiwan University, Taipei, Taiwan; 3 Bionet Corp., Taipei, Taiwan; 4 Gavin Herbert Eye Institute, University of California Irvine Medical Center, Orange, California, United States of America; University of Reading, United Kingdom

## Abstract

**Background:**

Keratoplasty is the most effective treatment for corneal blindness, but suboptimal medical conditions and lack of qualified medical personnel and donated cornea often prevent the performance of corneal transplantation in developing countries. Our study aims to develop alternative treatment regimens for congenital corneal diseases of genetic mutation.

**Methodology/Principal Findings:**

Human mesenchymal stem cells isolated from neonatal umbilical cords were transplanted to treat thin and cloudy corneas of lumican null mice. Transplantation of umbilical mesenchymal stem cells significantly improved corneal transparency and increased stromal thickness of lumican null mice, but human umbilical hematopoietic stem cells failed to do the same. Further studies revealed that collagen lamellae were re-organized in corneal stroma of lumican null mice after mesenchymal stem cell transplantation. Transplanted umbilical mesenchymal stem cells survived in the mouse corneal stroma for more than 3 months with little or no graft rejection. In addition, these cells assumed a keratocyte phenotype, e.g., dendritic morphology, quiescence, expression of keratocyte unique keratan sulfated keratocan and lumican, and CD34. Moreover, umbilical mesenchymal stem cell transplantation improved host keratocyte functions, which was verified by enhanced expression of keratocan and aldehyde dehydrogenase class 3A1 in lumican null mice.

**Conclusions/Significance:**

Umbilical mesenchymal stem cell transplantation is a promising treatment for congenital corneal diseases involving keratocyte dysfunction. Unlike donated corneas, umbilical mesenchymal stem cells are easily isolated, expanded, stored, and can be quickly recovered from liquid nitrogen when a patient is in urgent need.

## Introduction

There are growing interests in the use of stem cells, e.g., embryonic stem cells, mesenchymal stem cells, hematopoietic stem cells, etc. in treating human congenital diseases caused by genetic mutation and acquired diseases [1]–[8]. Especially, transplantation of mesenchymal stem cells has drawn much attention in regenerative medicine in the past decade [9], [10]. Since mesenchymal stem cells (MSCs) are first isolated from bone marrow, these plastic adherent cells have been identified and cultivated from many connective tissues, e.g., umbilical cord, amniotic membrane, cartilage, adipose tissue, and conjunctiva [11]–[14]. MSCs are multipotent and are capable of differentiating into various progenitor cells that form connective tissues such as bone, bone marrow, cartilage, adipose tissue, etc. Thus, there have been growing interests in the application of MSC transplantation in regenerative medicine to repair and restore normal function of diseased and injured tissues. For example, bone marrow MSCs have been used in treating diseases in animal models as well as in human clinical trials in areas such as autoimmune diseases, solid organ allograft survival, hepatic cirrhosis, kidney diseases, neuro- and muscle-degenerative diseases, myocardial infarction, and spinal cord injury [15]–[21]. Reports using stem cells isolated from individuals have demonstrated that the number of cells as well as the differentiation and proliferation potential of MSCs decreases with the donors' age [22], [23]. Umbilical cord-derived mesenchymal stem cells (UMSCs) from newborns are presumably young and therefore are likely to have a more potent proliferation and differentiation capability. Other advantages to the use of UMSCs is that the availability of umbilical cords from newborn babies is almost unlimited and the cells can be expanded and stored in liquid nitrogen in a tissue bank and thawed for use. However, the utilization of human UMSCs in treating disease is surprisingly rare in comparison to the use of bone marrow MSCs. Only a limited number of publications have reported on the success of using human UMSCs to treat liver fibrosis and spinal cord injury in animal models [24]–[27].

Dr. Eduard Zirm performed the first corneal transplantation in 1905 and to date this technique remains the most effective way for treating corneal blindness caused by microbial infection, mechanical and chemical injuries, and congenital defects due to genetic mutation [28]. For example, trachoma is the number one cause of corneal scarring worldwide, particularly in developing countries where hygiene and public health services are inadequate. In developed countries, pseudophakic bullous keratopathy, congenital hereditary corneal defect, e.g., congenital corneal stromal dystrophy, corneal lattice dystrophy, keratoconus, etc., and corneal injuries are frequent corneal diseases requiring corneal transplantation [29]. In the United States, there are only enough donated corneas suitable for transplantation for the patients in the direst need; however, this number is expected to decrease as the number of individuals receiving LASIK (laser in situ keratomileusis) increases. Worldwide, there is a shortage of suitable corneas for transplantation because of religious and/or regional customs. Corneal blindness in developing countries is exacerbated by the shortage of trained medical personnel (e.g., qualified corneal surgeons) and adequate medical facilities to perform corneal transplantation. Thus, many children do not receive proper treatment leading to permanent visual impairment that can ultimately lead to a substantial burden on both the family and society as a whole. It should be noted that currently there is no effective alternative procedure in lieu of keratoplasty in treating corneal blindness. Therefore, it is necessary to develop alternative treatment regimens to replace conventional corneal transplantation, which can be performed in the suboptimal medical conditions found in developing countries as well as under emergency situations, e.g., battle field, disastrous regions, earthquake, etc. One such alternative treatment is the transplantation of multipotent MSCs.

Lumican and keratocan, belonging to the family of small leucine rich proteoglycans, are the major keratan sulfate proteoglycans in the corneal stroma, which are required for corneal transparency [30]. Lumican null (*Lum^-/-^)* mice manifest thin and cloudy corneas due to disorganization of stromal extracellular collagen matrix and down-regulated expression of keratocan [31], [32]. In this study, we used *Lum^-/-^* mice as an experimental model to test the efficacy of UMSCs transplantation in treating congenital corneal diseases. Our results indicate that human UMSCs transplantation greatly improved corneal stromal thickness and transparency in *Lum^-/-^* mice with little or no host graft rejection.

## Results

### Characteristics of UMSCs isolated from human umbilical cord

**Figure 1:**
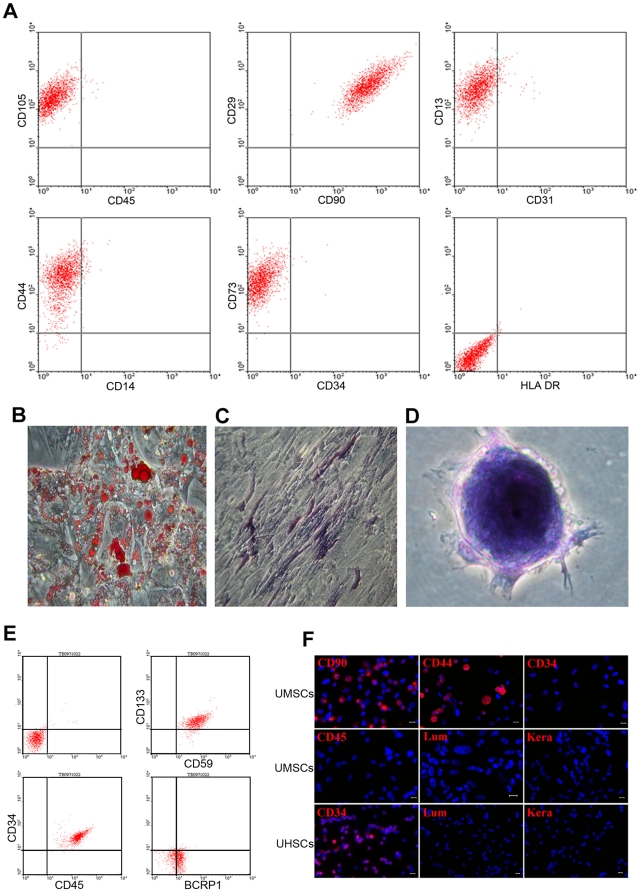
FACS analysis of UMSCs and UHSCs. **A.** UMSCs isolated from human umbilical cord were characterized by flow cytometry using CD markers; and these plastic adherent cells were positive to CD105/SH, CD13, CD29, CD44, CD73/SH3, and CD90, and negative to CD34, CD14, CD45, CD31, and HLA-DR (MHC-II). **B.** There were large amounts of lipid droplets (red) in UMSCs shown by Oil-red O staining after adipogenesis induction. **C.** Under osteogenic culture conditions, UMSCs expressed alkaline phosphatase (blue). **D.** Toluidine blue staining revealed that UMSCs synthesized chondroitin sulfate after the induction of chondrogenesis. **E.** UHSCs were positive to CD34, CD45, CD59, CD133, and BCRP1, which were identified by flow cytometry. **F.** Immunofluorescent staining also revealed that UMSCs were positive to CD90, CD44, and negative to CD34, CD45; UHSCs were positive to CD34. Neither UMSCs nor UHSCs expressed keratocan and lumican before the transplantation. Scale bars: 10 µm; Blue: DAPI.

Two populations of stem cells, UMSC and umbilical cord-derived hematopoietic stem cells (UHSCs) were isolated from human umbilical cord and cord blood, respectively. To characterize the stem cells, flow cytometry was used to determine the expression pattern of CD markers. The results showed that >95% of the fibroblast-like plastic-adherent cells expressed characteristic CD markers of mesenchymal stem cells, e.g., CD105/SH2 (endoglin), CD73/SH3 (NT5E), CD90 (Thy1), CD29 (integrin β1), CD44 (HA receptor), CD13 (alanine aminopeptidase); and <1% of cells were positive for hematopoietic cell CD markers, e.g., CD45 (leukocytes), CD34 (hematopoietic stem cells), CD14 (co-receptor of TLR4 and MD-2), HLA-DR (MHC-II), and CD31 (endothelial cells) (**Fig. 1A**).

The plastic adherent UMSCs were multipotent and capable of differentiation into adipocytes, osteoblasts and chondrocytes when they were cultured in adipogenic, osteogenic, and chondrogenic medium, respectively [27], [33], [34]. To demonstrate differentiation into these cell types, staining with oil-red O, alkaline phosphatase, and toluidine blue were performed. Oil-red O staining showed large amounts of lipid droplets indicating induction of adipogenesis (**Fig. 1B**). Osteogenesis was confirmed by expression of alkaline phosphatase (**Fig. 1C**) while the synthesis of chondroitin sulfate verified by toluidine blue staining demonstrated induction of chondrogenesis (**Fig. 1D**).

To assess the percentage of CD34 positive cells after the volume reduction of cord blood, flow cytometry was employed to examine the characteristics of cord blood [35]. The results showed that the cord blood cells were positive to CD34, CD45, CD59, CD133, and BCRP1 (**Fig. 1E**).

To verify the expression pattern of UMSCs and UHSCs prior to transplantation, immunofluorescent staining was performed with selected CD markers for MSCs and HSCs, as well as to keratocan and lumican. The results showed that both human UMSCs and UHSCs did not express keratocan and lumican, and UMSCs were CD34 negative (**Fig. 1F**).

### Corneal stromal transplantation of UMSCs increased stromal thickness and decreased corneal haze in *Lum^-/-^* mice

**Figure 2:**
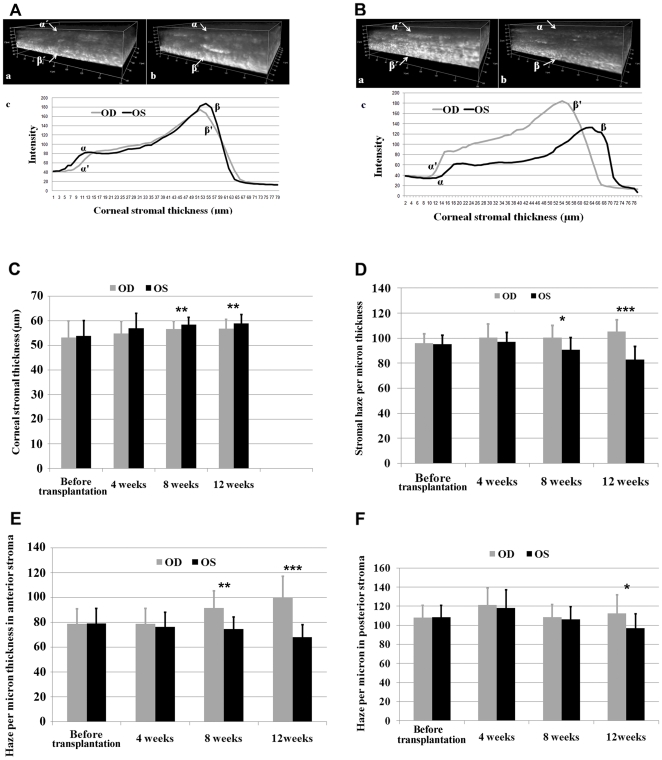
Corneal stromal thickness and transparency in *Lum^-/-^* mice was improved by the transplantation of UMSCs. **A.** Before UMSC transplantation, there were similar levels of corneal stromal light scattering and thickness in the right (OD, a) and left (OS, b) eye. Panel c contains histograms of light scattering and stromal thickness. **B.** Panel a and b represented the 3-dimensional images of HRT examination 12 weeks after UMSCs transplantation; and clearly, lower light scattering and increased thickness displayed in the transplanted corneal stroma (b) than that of an untransplanted cornea (a). The histograms showed thicker and less light scattering (more transparent) in the stroma of transplanted corneas than those of untransplanted corneas after 12 weeks. **C.** Corneal stromal thickness was significantly increased in the transplanted cornea than that of the untransplanted cornea after 8 weeks. **D.** Compared to the untransplanted cornea the average light scattering significantly decreased in the transplanted cornea 8 and 12 weeks after UMSC transplantation. In untransplanted corneas, there is an increase of light scattering with age. **E.** The average pixel intensity per µm of light scattering in the anterior corneal stroma significantly decreased after 8 and 12 weeks. **F.** The improvement of light scattering as measured by average pixel intensity in the posterior stroma was not significantly improved until 12 weeks after transplantation. (n = 11; * *p<0.05*; ** *p<0.01*; *** *p<0.001*).

To examine the possibility whether human UMSC transplantation can be used as a treatment regimen for congenital corneal diseases of genetic mutation, UMSCs were transplanted into the left corneas (OS) of 6 week-old *Lum^-/-^* mice (n = 11), the right corneas (OD) served as contra-lateral controls. In vivo confocal microscopy with the Heidelberg Retinal Tomograph-HRTII Rostock Cornea Module (Heidelberg Engineering GmbH, Heidelberg, Germany) was employed to capture through-focus/serial images by a continuous Z-axis laser scanning of the entire corneal stroma starting at the corneal epithelium and ending at the endothelium, at various time points including before transplantation and 4, 8, and 12 weeks after transplantation. Five different regions of each corneal center were scanned. Corneal stromal haze was determined by measuring total pixel intensity of light scattering through the 3D volume while corneal stromal thickness was measured by calculating the axial distance from the anterior to posterior stroma, as previously described [36]. **Fig. 2A and 2B** respectively displayed 3-dimensional corneal images of a *Lum^-/-^* mice before and 12 weeks after transplantation. It is clear to see that less corneal haze and thicker stroma were observed in UMSC transplanted corneas. Through calculating corneal stromal depth by Z-stacks from in vivo confocal microscopy, stromal thickness significantly increased in the corneas transplanted with UMSCs as early as 8 weeks after surgery (**Fig. 2C**). Although there was only an average of a 4.1 micron increase in corneal stromal thickness, this represents approximately a 10% increase when compared to the full stromal thickness of un-transplanted corneas. It should be noted that transplantation of human UHSCs failed to improve the corneal transparency and stromal thickness in *Lum^-/-^* mice (data not shown).

To more closely assess the changes of stromal light-scattering, total pixel intensity of 3-dimensional volume was divided by stromal thickness and the average of light scattering per micron thickness of the corneal stroma was calculated. In untransplanted corneas, light scattering in the corneal stroma showed a slight increase with age; in contrast, corneal light scattering decreased in transplanted corneas, which became statistically significant after 8 weeks (**Fig. 2D**).

Studies have shown that increased light scattering in the posterior stroma of *Lum^-/-^* mice is associated with the assembly of abnormal corneal collagen fibrils leading to increased fibril size and spacing [37]. To further analyze the effect of UMSCs transplantation on light scatting, corneal stromal volume was divided in half and the average pixel intensity per micron thickness of the anterior and posterior cornea calculated. The data indicate that the scattering increases in the anterior stroma with age, and that transplantation has a significant effect on this age-related scattering than the original. The results revealed a significant decrease in the anterior stroma 8 weeks after transplantation as compared to that of untreated corneas, while stromal haze was not significantly improved in the posterior until after 12 weeks (**Fig. 2E,F**). These findings suggest that in order to rescue severely disorganized corneal collagen as is found in the posterior stroma of *Lum^-/-^* mice may require longer than 12 weeks. Transplantation of UHSCs failed to improve the transparency and stromal thickness of *Lum^-/-^* corneas (data not shown)

### Stromal collagen of *Lum^-/-^* mice was re-organized through the transplantation of UMSCs

**Figure 3:**
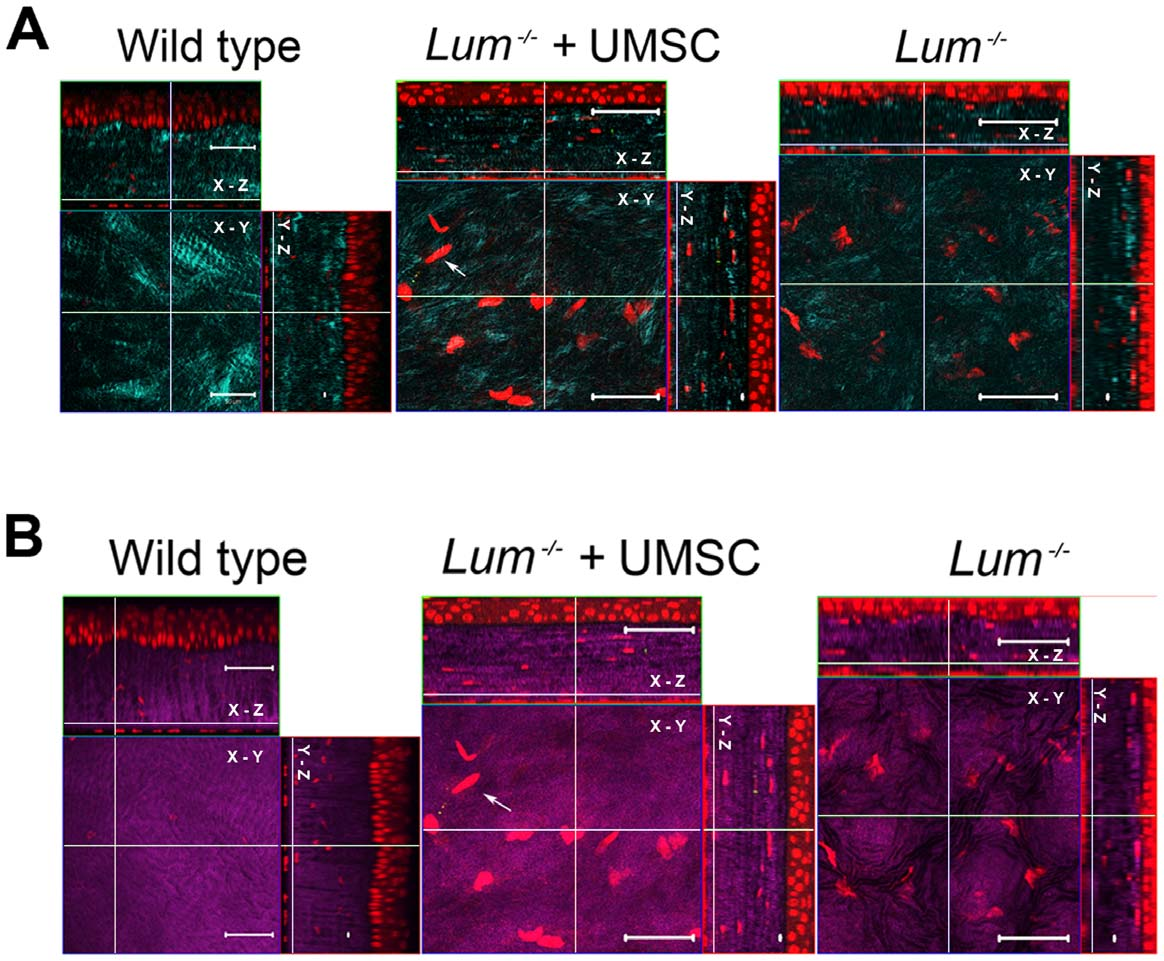
Improved collagen fiber organization in the corneal stroma of *Lum^-/-^* mice 12 weeks after UMSC transplantation. **A.** The SHG forward scattering images revealed that collagen fibers (cyan) were more regularly organized in the transplanted cornea (n = 5) than that of the untransplanted cornea (n = 2), especially in the middle and posterior stroma; moreover, the size of collagen fibers was larger in the transplanted cornea than that of the untransplanted cornea. **B.** The back scattering images showed more flattened images in the transplanted cornea and uneven lamellae (magenta) in the untransplanted cornea. Left panels, wild type mouse cornea; middle panels, *Lum^-/-^* cornea transplanted with UMSCs; right panels, untransplanted *Lum^-/-^* cornea. White arrows: UMSCs; Red: Syto59; Scale bars: 50 µm.

Non-linear optical imaging (NLO) of second harmonic generated signals (SHG) from collagen is a novel method for assessing collagen matrix organization in normal and diseased corneas [38]. To determine whether the improvement of corneal transparency is associated with a change in the organization of collagen fibers in *Lum^-/-^* mice after UMSCs transplantation, 5 transplanted and 2 un-transplanted corneas from *Lum^-/-^* mice were stained with Syto 59 and subsequently examined by NLO microscopy using the methods previously described [39]. In comparison to wild type corneas, the images of forward-scattered SHG signals revealed that longer, larger, and more organized collagen fibers were present in the transplanted posterior corneal stroma compared to that of un-transplanted corneas, albeit the structure of the collagen matrix did not fully recover to that of wild type corneas. For example, the wide bundles of collagen fibers that form the wide posterior collagen lamellae in the wild type cornea were completely disrupted in the *Lum^-/-^* mouse (**Fig. 3A**). By comparison, UMSC transplanted corneas showed a partial recovery with longer and more intact collagen fibers compared to the *Lum^-/-^* mice but not as broad as in the wild type cornea. This suggests that more time or transplantation into the posterior stroma may be required to get full restoration of corneal transparency in these corneas. Similarly, images from back scattered SHG signals also showed better organized collagen lamellae in the posterior stroma of wild type mice, which was completely disrupted in the *Lum^-/-^* mouse (**Fig. 3B**). Transplantation of UMSCs also appeared to partially restore this organized pattern.

Taken together, our findings suggest that a xenograft of human UMSCs into the mouse cornea was capable of improving corneal transparency and stromal thickness in *Lum^-/-^* mice. It is worthy to note that the success of this therapeutic strategy may depend on several factors: 1. improvement of cell function by UMSCs transplantation; 2. survival of UMSCs in the new tissue environment; 3. differentiation of UMSCs into specific cell types of the target tissue; 4. maintenance under the same cell cycle control as the host tissue without causing tumorigenesis. Herein, these results revealed that UMSCs transplantation can improve corneal transparency and stromal thickness through the improvement of the collagen matrix. Therefore, the following experiments were performed to determine whether UMSCs transplanted into *Lum^-/-^* mice corneas survive, differentiate and maintain cell cycle control similar to host keratocytes.

### Human UMSCs assume dendritic cell morphology in the mouse corneal stroma

**Figure 4:**
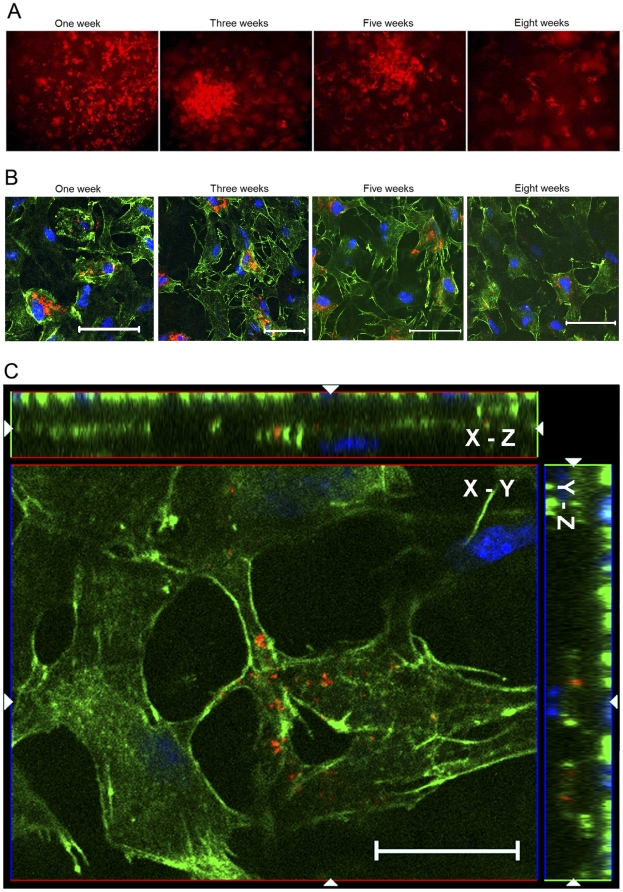
Transplanted UMSCs undergoing morphological changes resembling that of host dendritic keratocytes. **A.** The images of in vivo fluorescent stereoscopy showed that DiI-labeled UMSCs (red) gathered around the area of the injection tunnel and displayed a round cell shape within the first week following transplantation; later, the cells migrated out and became dendritic in shape. At 8 weeks, the cells were homogeneously distributed in the whole cornea. (magnification, 200×). **B.** Confocal microscopy revealed that transplanted UMSCs displayed a round-like cell shape by phalloidin staining with the whole mount cornea in the first week and afterwards the cells extended their protrusions and displayed a flat and dendritic cell shape (Scale bars: 50 µm; Blue: nuclear stain by DAPI). **C.** Following Phalloidin (green) staining with the whole mount *Lum^-/-^* mouse corneas 5 weeks after UMSCs transplantation, confocal images revealed that DiI-labeled UMSCs displayed a dendritic and flat cell shape and formed a three dimensional network between the host stromal cells and the donor cells via their extensive dendritic processes, which is similar to those of host keratocytes. Scale bar: 20 µm; Blue: nuclear stain by DAPI.

Human UMSCs in suspension appear small, have a round cell shape with a few cell processes, and have large and round nuclei with a prominent nucleolus [40]. For tracing the morphological changes of UMSCs in the corneal stroma, UMSCs were ex vivo labeled with DiI or DiO prior to transplantation into the cornea and examined by fluorescent stereomicroscopy. Initially, UMSCs stayed at the site of injection and then gradually migrated to the corneal periphery. Morphologically, UMSCs displayed a round cell shape in the first week after transplantation. Three weeks later, transplanted UMSCs were homogeneously distributed in the cornea and concurrently underwent morphological changes to assume a dendritic cell shape as revealed by fluorescent stereomicroscopy **(** **Fig. 4A** **)**. These morphological changes were better illustrated by confocal microscopy after phalloidin staining with the whole mount *Lum^-/-^* mouse cornea (**Fig. 4B**). Z-stack confocal images revealed that transplanted UMSCs after 5 weeks displayed a dendritic and flat cell shape and formed a three dimensional network of cell-cell contacts between host stromal cells and donor cells via their extensive dendritic processes, which is similar to those of native keratocytes (**Fig. 4C**).

### Survival and proliferation of transplanted UMSCs in mouse corneal stroma

**Figure 5:**
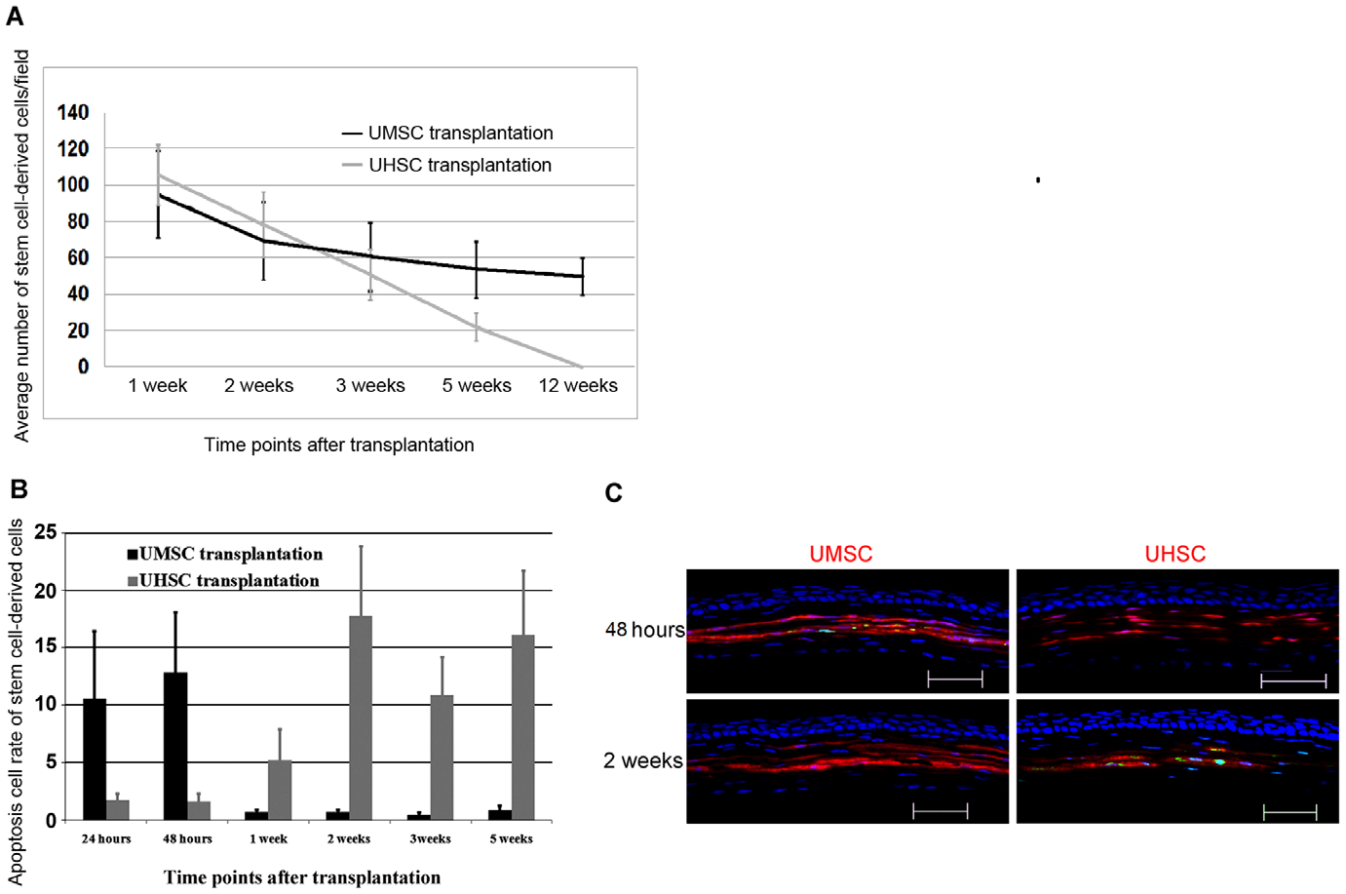
The apoptosis of transplanted UMSCs and UHSCs in the recipient *Lum^-/-^* corneal stroma. **A.** Two weeks after transplantation, the number of UHSCs and UMSCs decreased; afterwards, UHSCs continuously diminished and almost disappeared from the cornea after 5 weeks; however, UMSC numbers maintained at a relative constant level and more than half of UMSCs survived in the corneal stroma up to 12 weeks (n≥6 in each time point). **B** and **C.** TUNEL assay revealed that a large number of UMSCs was labeled (green) in 48 hours after transplantation, thereafter, very few apoptotic UMSCs could be detected after one week. In contrast very few apoptotic cells were seen in the UHSCs within the first 48 hours after the transplantation and the number of apoptotic UHSCs increased after 1 week and maintained at high levels thereafter (n≥4 in each time point). Scale bars: 50 µm; Blue: DAPI.

Successful xenograft of human UMSCs into the mouse cornea requires that the transplanted cells escape from host immune rejection and are able to continue to survive in the mouse corneal stroma. To compare the survival rates of UMSCs and UHSCs in the corneal stroma, UMSCs and UHSCs labeled by DiI or DiO were transplanted into the corneal stroma of *Lum^-/-^* mice. At different time points, the nuclei were stained with DAPI and the number of transplanted cells was determined by counting DiI or DiO-labeled cells in confocal Z-stack images. The results revealed that immediately after transplantation the number of UMSCs underwent a rapid decrease, leveling off by two weeks and of these cells half of them survived for more than 12 weeks in the stroma. By contrast, the number of transplanted UHSCs in recipient corneas rapidly and continuously decreased (**Fig. 5A**).

A TUNEL assay was performed to examine if apoptosis may account for the cell loss after transplantation. As shown in Figure 5B and 5C, the rate of apoptosis after UHSCs transplantation differed from that of UMSCs. The apoptotic rate of UMSC derived cells was initially higher than that of transplanted UHSCs within the first 48 hours of transplantation; however, this rate markedly decreased 1 week after transplantation (**Fig. 5B,C**). The decreased apoptotic rate of UMSC-derived cells was associated with a slower decrease in cell number after 3 weeks and 5 weeks. By contrast, UHSC-derived cells showed a marked increase in the rate of apoptosis after 1 week and remained high for up to 5 weeks (**Fig. 5B,C**), which is consistent with the continued decline in cell numbers noted earlier.

**Figure 6:**
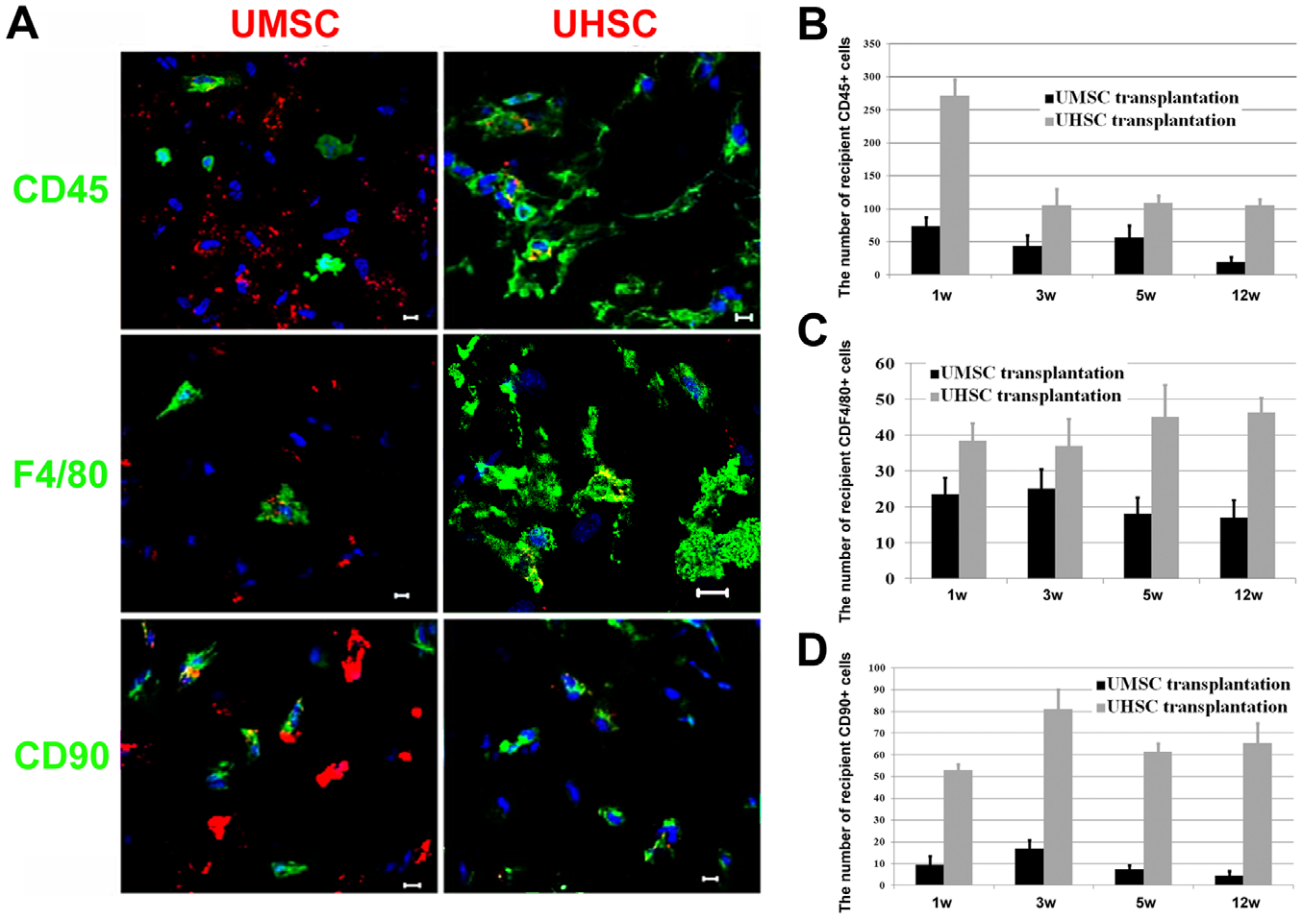
The recipient inflammatory response after UMSCs and UHSCs transplantation into *Lum^-/-^* cornea. **A.** Immunostaining with antibodies against inflammatory cells, e.g., CD45 (leukocyte), CD90 (T-Cell) and F4/80 (macrophage) demonstrated the presence of large numbers of immune and inflammatory cells in UHSC transplanted corneas but not in UMSC transplanted corneas, indicating that UMSCs might suppress the host immune response. **B.** More CD45+ leukocytes invaded UHSC-recipient corneal stroma after the transplantation and the peak of leukocyte infiltration occurred one week after transplantation (n≥5 in each time point). **C.** The recipient F4/80+ macrophages were present in corneas transplanted with UMSCs and UHSCs. In contrast, more macrophages were found in corneas with UHSC transplantation than that of UMSC transplantation (n≥5 in each time point). **D.** The infiltration of recipient CD90+ T-cells appeared at the first week and maintained at high levels after UHSC transplantation; very few CD90+ cells were seen after UMSCs transplantation (n≥5 in each time point). Scale bars: 10 µm; Blue: DAPI.

It has been reported that MSCs suppress the host immune response [41], which might explain why UMSCs survive in the mouse stroma; in addition to the fact that the cornea is an immune privileged tissue. Thus, immunofluorescent staining with anti-CD45, anti-CD90, and anti-F4/80 antibodies were used to determine the host immune and inflammatory response after UMSC and UHSC transplantation. The results showed that there were more CD45+ leukocytes, CD90+ (Thy-1 cells), and F4/80+ macrophages presented in UHSC transplanted corneas than those transplanted with UMSCs (**Fig. 6A–D**), indicating that UHSC transplantation triggered a strong host immune rejection.

**Figure 7:**
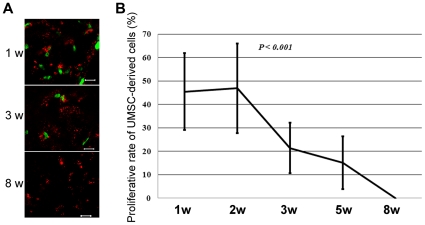
UMSCs-derived cells changed to be quiescent in *Lum^-/-^* cornea after the transplantation. **A.** BrdU labeled cells were identified by whole mount immunostaining with anti-BrdU antibody (green) and the results revealed that BrdU-labeled UMSCs (red) presented at 1 and 3 weeks after transplantation, no BrdU-labeled cells were found in corneas 8 weeks after transplantation. **B.** The numbers of BrdU-labeled UMSCs decreased 2 weeks after transplantation; by eight weeks, there were no BrdU-labeled cells found in the stroma, suggesting that transplanted UMSCs became quiescent. n = 6 for each time point and condition. Scale bars: 10 µm; Blue: DAPI.

It should be noted that the fluorescence intensity of individual DiI- or DiO-labeled UMSCs appeared to decrease slightly over time (**Fig. 4B**), suggesting that transplanted UMSCs proliferate. To verify this possibility, BrdU labeling was performed 2 hours prior to sacrifice. The corneas were then subjected to whole mount immunostaining with an anti-BrdU antibody. As expected, transplanted UMSCs underwent proliferation evidenced by the presence of BrdU-positive cells within the first few weeks after transplantation (**Fig. 7A,B**). The transplanted UMSCs became quiescent (no BrdU positive UMSCs) 8 weeks after transplantation, a characteristic feature of adult corneal keratocytes.

### Expression of keratocyte markers by UMSCs transplanted into the corneal stroma

**Figure 8:**
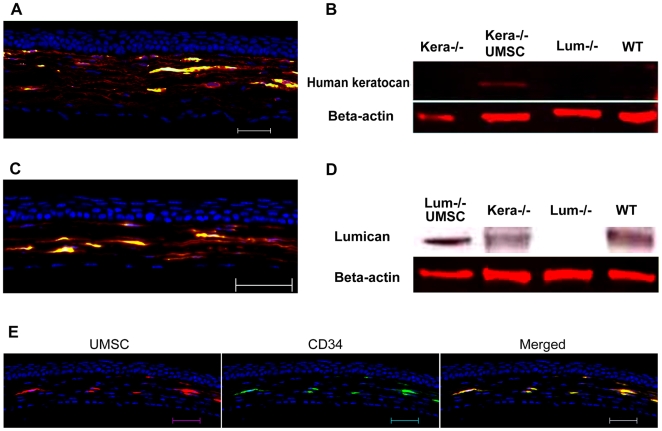
The synthesis of KS-keratocan, KS-lumican, and expression of CD34 by transplanted UMSCs. **A.** Immunostaining with anti-human keratocan antibody displayed that keratocan (red) was distributed surrounding the transplanted UMSCs (green) in the anterior stroma of the *Kera^-/-^* mouse. **B.** Western blot revealed that human keratocan (45 kD) was detected after enzymatic digestion to remove keratan sulfate glycosaminoglycan (KS-GAG) in the *Kera^-/-^* cornea transplanted with UMSCs, but not in untransplanted corneas of *Kera^-/-^*, *Lum^-/-^* and wild type mice. n = 5. **C.** Immunostaining with anti-mouse lumican antibodies (cross reacts to both human and mouse lumican) displayed that lumican (red) presented in *Lum^-/-^* mouse cornea after UMSC (green) transplantation. **D.** Western blot revealed that lumican (45 kD) was detected after enzymatic removal of KS-GAG in *Lum^-/-^*, *Kera^-/-^*, and wild type mouse corneas transplanted with UMSCs, but not in the *Lum^-/-^* mouse cornea (n = 4). **E.** Immunostaining revealed that transplanted human UMSCs (red) were stained by anti-CD34 (green) in *Lum^-/-^* mouse corneas six weeks after transplantation. Scale bars: 50 µm; Blue: DAPI.

As mentioned above, one of the criteria for successful cell therapy is that donor cells must differentiate and assume a native (host) phenotype and express genes specific to the target cells. To verify that UMSCs could indeed assume a keratocyte phenotype, the synthesis of KS-lumican and KS-keratocan was determined. We have previously generated a keratocan null (*Kera^-/-^)* mouse, which does not express keratocan, a cornea-specific keratan sulfate proteoglycan, and displays a thinner corneal stroma than that of a wild type mouse [42]. For these studies, we took advantage of keratocan and lumican null mice whose expression of keratocan and lumican by UMSCs could be determined by immunofluorescent staining and western blot analysis. **Fig. 8A** shows the presence of keratocan in the corneal stroma of *Kera^-/-^* mice detected by rabbit anti-human keratocan antibody 5 weeks after transplantation of UMSCs. Western blot analysis indicated that a single 45 kD protein was detected after treatment with keratanase and/or endo-β-galactosidase (**Fig. 8B**). These observations are consistent with the notion that the newly synthesized human keratocan expressed by transplanted UMSCs is keratan sulfate proteoglycan. Similar results were obtained when UMSCs were transplanted into corneas of *Lum^-/-^* mice (**Fig. 8C,D**). Immunostaining with anti-CD34 antibodies, a known marker of human keratocytes [43], [44], was also performed. **Fig. 8E** demonstrated that transplanted human UMSCs in the stroma were CD34-positive 5 weeks after transplantation.

### Improved host keratocyte function by UMSCs transplantation in *Lum^-/-^* mice

**Figure 9:**
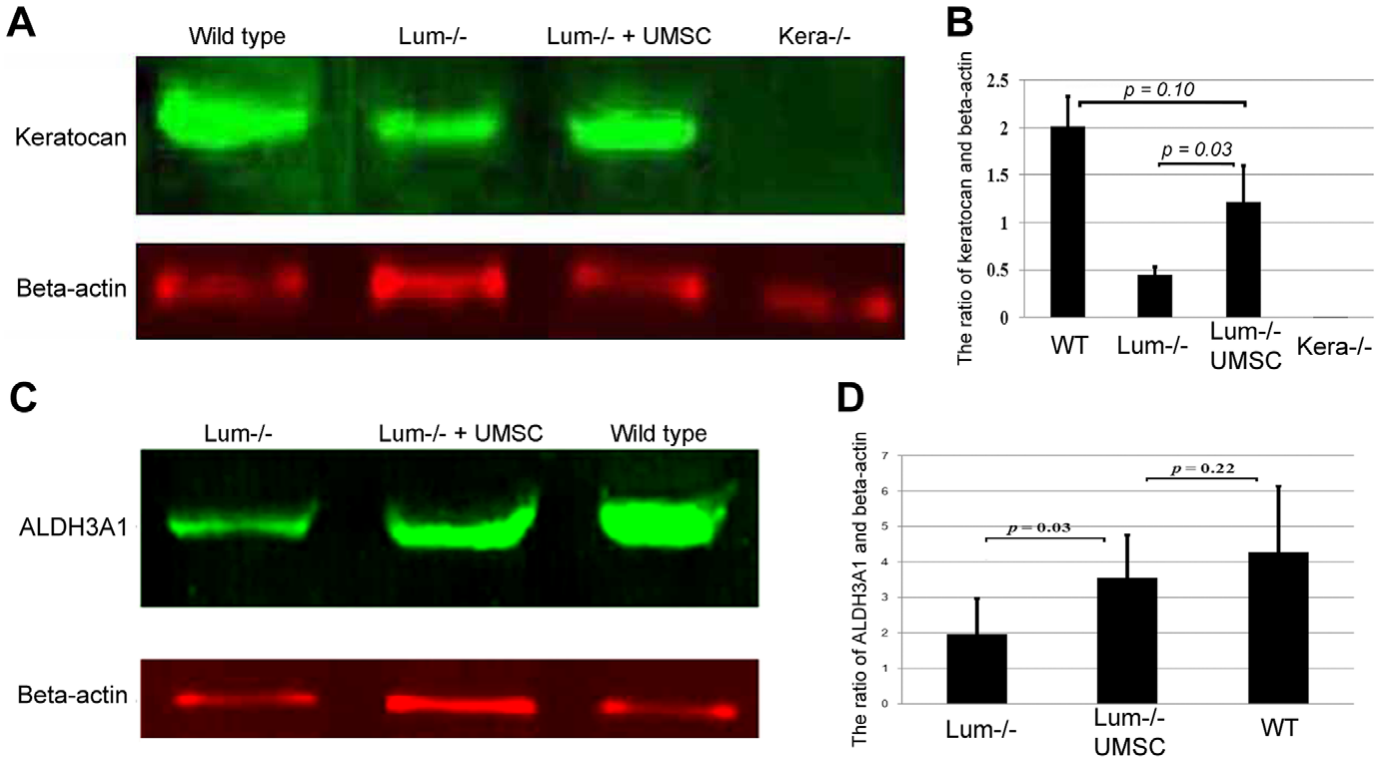
Up-regulation of keratocan and ALDH3A1 was observed in the *Lum^-/-^* mouse cornea after UMSC transplantation. Semi-quantitative Western blot analysis was performed to determine increased synthesis of keratocan and aldehyde dehydrogenase by recipient keratocytes in corneas transplanted with UMSCs. Panels **A** and **B** indicated that the keratocan expression level significantly increased after UMSC transplantation compared to untransplanted corneas. Panels **C** and **D** showed that the expression of ALDH3A1 was significantly increased in *Lum^-/-^* mice after UMSC transplantation (n = 4).

Our previous studies suggest that lumican is a matrikine and plays a pivotal role in regulating multiple cellular functions, e.g., cell migration, cell proliferation, survival, and gene expression in addition to serving as a regulator of collagen fibrillogenesis [30], [45], [46]. Thus, it is likely that UMSCs transplantation into *Lum^-/-^* corneas may improve overall tissue function by up-regulating keratocyte specific genes, i.e., keratocan and aldehyde dehydrogenase that are down regulated in *Lum^-/-^* corneas [45]. To examine this possibility, semi-quantitative western blot analysis was employed to determine the levels of keratocan and aldehyde dehydrogenase expression in *Lum^-/-^* mouse corneas 5 weeks after UMSC transplantation. **Fig. 9A–D** show that keratocan and aldehyde dehydrogenase levels were increased 2–3 folds after UMSC transplantation.

## Discussion

Although penetrating keratoplasty (corneal transplantation) has been the most successful organ transplantation, the supply of donor corneas suitable for transplantation is diminishing because of the increased popularity of laser refractive surgery, which leaves the cornea unsuitable for later organ transplantation. Thus, alternatives to organ transplantation for treating corneal blindness are needed. Use of keratoprostheses has achieved some success as one of the final solutions when an ordinary keratoplasty is not possible [47], [48]. Recently, it has been demonstrated that transplantation of human corneal stromal stem cells can improve corneal transparency in *Lum^-/-^* mice [49]. It should be noted however that the number of corneal stromal stem cells that can be obtained from human corneas is quite limited and very technically demanding.

The present study is the first to provide the evidence demonstrating that UMSCs transplantation improved corneal transparency and stromal thickness in *Lum^-/-^* mice. The observed increase in corneal function appears to be associated with improved organization of the collagen matrix when compared to that of untransplanted *Lum^-/-^* corneas. We have subsequently provided data demonstrating that transplanted UMSCs: can survive and escape host rejection, showing a reduced host infiltration of immune cells; assume a keratocyte-like phenotype, having a dendritic morphology that forms a three dimensional network and cell-cell contacts with host stromal cells; and acquire a unique gene expression pattern similar to that of corneal keratocytes, e.g., synthesis of KS-keratocan and KS-lumican, aldehyde dehydrogenase, and CD34 and became quiescent after 8 weeks.

Further studies are certainly needed to elucidate molecular and cellular mechanisms that govern the differentiation of UMSCs within the stroma of recipient *Lum^-/-^* mice. For example, does the improved organization of the collagen matrix in treated *Lum^-/-^* corneas require de novo synthesis of collagen or is the presence of human lumican produced by the donor cells sufficient? Our studies indicated that transplantation of human UHSCs elicited a significant immune response, which coincided with the loss of transplanted UHSCs within a few weeks of transplantation. In contrast, transplantation of human UMSCs into mouse corneas caused little or no host immune response. The molecular and cellular mechanisms by which MSCs suppress the host immune response are not known, albeit co-transplantation of MSCs has been widely used in regenerative medicine [50], [51]. Likewise, bone marrow derived mesenchymal cells have been co-transplanted with bone marrow cells in cancer patients and other solid organ transplantation to reduce graft versus host reaction [52]. It has been recently demonstrated that bone marrow MSCs produce nitrous oxide (NO) when stimulated by leukocyte cytokines, which then inhibits T-cell proliferation [53]. Thus, it is possible that a similar mechanism is involved in UMSCs survival. Interestingly, there was a high rate of UMSCs apoptosis in the first 48 hours after transplantation with very low apoptosis after 1 week. We propose that the early encounter of UMSCs with cytokines produced by inflammatory cells causes UMSCs apoptosis shortly after transplantation, and subsequently up-regulates the expression of iNOS (inducible nitrous oxide synthetase and the production of NO that in turn suppress T-cell proliferation. It should be noted, however, the mechanism that triggers UMSC apoptosis in the early phase of transplantation is not known. It is possible that heterogeneity of UMSCs population may account for the observation. Nevertheless, further studies are needed to test these possibilities.

Taken together, our data showed that xenograft of human UMSCs into mouse corneas did not trigger graft rejection. Thus, allograft of human UMSCs into human corneas is likely to be tolerated and is of great potential for being successful in treating human corneal blindness. Therefore, cell based therapy with umbilical mesenchymal stem cells is a promising medical procedure to treat congenital corneal diseases involving keratocytes dysfunction.

Based on the data provided, we suggest that UMSC transplantation may be used as an alternative treatment to corneal transplantation for genetic and acquired corneal diseases. Unlike donated corneas, the supply of human UMSCs is almost unlimited. They are easily isolated from umbilical cord and can be expanded in cell culture, and can be stored by tissue banking and quickly recovered-from liquid nitrogen in preparation for transplantation. In addition, intrastromal injection is a simple surgical procedure and can be performed by adequately trained medical personnel under even suboptimal medical conditions in developing countries, areas of natural disaster, and/or the battle field.

## Materials and Methods

### Isolation and culture of umbilical cord derived UMSCs

After disinfection with 75% ethanol, the fresh umbilical cord was transferred to PBS where excess blood on the surface was removed. After removal of blood vessels, the tissue was sliced into very small pieces followed by treatment with 0.05% trypsin (Gibco, Carlsbad, CA) and 300U collagenase (Stem Cell Technologies) in alpha-MEM (Gibco) for 1 hour at 37°C. The solid parts were filtered and the filtrate was centrifuged at 400G to collect the cells. After removing the supernatant, the cells were cultured in alpha-MEM supplemented with 10% fetal bovine serum (Hyclone, Waltham, MA) in 5% CO_2_ in a 37°C incubator. On the next day, non-adherent cells were removed. The medium was changed every 3–4 days until the plastic adherent cells reached to near confluence. At this point, the cells were harvested with Trypsin/EDTA and subcultured at a density of 3–6×10^3^ cells/cm^2^

According to the current regulation of the Department of Health of Taiwan, only parent's consent is required for the collection of umbilical cords from the newborn baby. Such parents' written consent forms acknowledging tissue donation were obtained prior to the collection of umbilical cords. In the present studies, no human subject was involved, thereby no IRB is required. However, the animal protocol (#Kao 05-01-11-02) for the use of these cells in experimental mice were reviewed and approved on February 11, 2008 by IACUC of the University of Cincinnati.

### Characteristics of human UMSCs by flow cytometry

Fluorescein isothiocyanate (FITC)-conjugated or phythoerythrin (PE)-conjugated antibodies against CD14, CD34, CD45, CD44, CD73, CD90, CD166, HLA-ABC, HLADR (BD Biosciences, San Jose, CA) and CD105 (Southern Biotech) were used. The plastic adherent cells were detached with trypsin/EDTA in PBS and incubated (30 min) with the antibodies. In each case, 1×10^4^ events were acquired and analyzed with Cell Quest software (Becton Dickinson, Franklin Lakes, NJ).

### *In vitro* UMSC differentiation

The plastic adherent cells were seeded at a density of 1×10^5^ cells/well (6-well plate) and incubated overnight. Osteogenic differentiation was induced using the STEMPRO® Osteogenesis Differentiation Kit (Gibco). Medium was changed twice a week over a period of 14∼21 days. To visualize osteogenic differentiation, cells were stained for alkaline phosphatase. Adipogenic differentiation was induced using the STEMPRO® Adipogenesis Differentiation Kit (Gibco). Induction medium was changed every 3–4 days and cells were cultured for at least 21 days. To demonstrate the presence of adipocytes-like cells, cells were fixed with 10% neutral buffered 3.7% formalin for 10 min, and then cytoplasmic inclusions of neutral lipids were stained with oil-red O (3 mg oil red/ml 60% isopropanol) for 15 min. Chondrogenic differentiation was induced using the STEMPRO® Chondrogenesis Differentiation Kit (Gibco) for 14 days and toluidine blue staining was employed to visualize proteoglycans.

### Cell processing prior to transplantation

Following the thawing of UMSCs from liquid nitrogen in a 37°C water bath, UMSCs were washed by centrifugation and resuspended in Dulbecco's modified eagle medium (DMEM, Invitrogen Corporation, Grand Island, New York). UMSCs were labeled by incubation in 1∶200 diluted DiO (green fluorescence) in DMEM for 3 hours at room temperature or DiI (red fluorescence) in DMEM for 30 min at 37°C. After washing, UMSCs were resuspended in PBS and a10 µl of cell suspension was mixed with an equal volume of 0.4% trypan blue to determine the total number of viable cells using a hemacytometer. The fluorescent intensity might decrease, if the labeled cells underwent proliferation.

### Intrastromal injection of stem cells

To determine the stromal haze and thickness, mice (n = 11) were anesthetized by intraperitoneal injection of ketamine hydrochloride (80 mg/kg) and xylazine (10 mg/kg). The eyes were rinsed with PBS and topically anesthetized with a drop of proparacaine. A corneal intrastromal tunnel into the stroma was created with a 33-gauge needle. 1×10^4^ cells in 2 µl PBS were injected into each cornea. After injection, the eyes were treated once with ophthalmic antibiotic ointment. For immunohistochemistry and western blot analysis, the numbers of mice used for each condition and time point were indicated in legends of individual figures.

### Analysis of corneal stromal haze and thickness

In vivo analysis of corneal stromal thickness and haze was performed with Heidelberg Retinal Tomograph-HRTII Rostock Cornea Module (HRT-II, Heidelberg Engineering Inc., Germany) according to the manufacturer's instruction. Briefly, a drop of GenTeal® Gel (Novartis Pharmaceuticals Corp., East Hanover, New Jersey) was applied to the tip of the HRT-II objective as immersion fluid. Subsequently, a series of images were collected to cover the whole stromal thickness as a continuous z-axis scan through the entire corneal stroma at 1–3 µm increments starting from the basal layer of corneal epithelium and ending at the corneal endothelium (the lens of HRTII has a working distance of 77 µm). A depth intensity profile from scans was generated by plotting the average pixel intensity per plane as a function of corneal depth. Corneal light scattering was then measured as previously described [54] by calculating the total pixel intensity as measured by the area under the curve. Corneal thickness was determined by measuring the axial distance from the anterior to posterior cornea.

### Fluorescence and non-linear optical imaging of second harmonic generation (SHG)

Briefly, following nuclear staining with Syto 59, the corneal button was mounted onto the slide with Mowiol medium (Calbiochem, La Jolla, CA) and imaged using a Zeiss Axiovert 200 inverted microscope platform with an 40×water immersion objective lens (numerical aperture, 1.2). The fluorescent signal detection from DiO and Syto 59 were obtained using 488 nm and 633 nm laser lines from the argon and red He-Ne lasers, respectively. Emitted light was detected using a 500 nm to 550 nm and 650 LP filters for green and Syto 59, respectively. Non-linear optical imaging of second harmonic generated signals (SHG) of collagen organization was performed using mode-locked titanium (MaiTai; Spectra-Physics Lasers Division, Mountain View, CA). The SHG forward-scattered signals were collected using a 0.8 NA condenser lens with a narrow band-pass filter (400/50) placed in front of the transmission light detector. Backward scattered SHG signals were detected with the Meta detector on the Zeiss LSM510 confocal microscope.

### BrdU administration and tissue preparation for morphology

Two hours prior to euthanasia the mice (n = 16) were given an i.p. injection of 80 mg/kg BrdU (Sigma) in PBS. Following euthanasia and the eyes were enucleated and fixed in 4% paraformaldehyde in 0.1 M phosphate buffer pH 7.4 (PB).

### Staining of cryosections and whole mount tissues

For conventional immunostaining, cryosections (10 µm thickness) were used. For the whole mount immunostaining, corneas were incubated with conjugated antibody in 0.3% Triton X-100 buffered solution overnight; for whole mount BrdU immunostaining, the whole cornea was treated by 4N HCl for 30 min, washed with PBS at room temperature, and then incubated with conjugated anti-BrdU antibody in 0.3% Triton X- 100 buffered solution overnight. After incubation, corneas were washed in 0.02% Triton X-100 in PBS for 3 to 6 hours followed by washing in PBS overnight. Finally, the corneas were mounted on slides with Mowiol medium for confocal laser scanning microscopy.

### Western blotting

A cornea was cut into small pieces under a stereomicroscope and suspended in 100 µl alkali extraction buffer containing 250 u/ml Benzonase nuclease (Novagen, WI) and 1X protease inhibitor cocktail (Sigma-Aldrich, St. Louis, MO) in 50 mM Tris (NaOH) pH 12 at 4°C overnight. After being clarified by centrifugation, the supernatant was neutralized with 10 mM Tris/HCl (pH = 6.0) to pH 7–8 and digested in 0.5 u/ml Keratanase at 37°C overnight. The protein was separated on a 4–20% SDS-PAGE mini gel and then transferred to an immobilon transfer membrane (Millipore Corp., Bedford, MA). The membrane was subjected to western blot analysis as described previously (45). Immune reactivity was visualized with DAB (secondary peroxidase conjugates) or with fluorescence imaging (secondary fluorescence conjugates) using Odyssey Infrared Imaging System (Li-COR Biosciences, Lincoln, Nebraska).

### Apoptotic analysis

Cryosections were processed by proteinase K solution and then fixed in 4% paraformaldehyde and then subjected to TUNEL assay with DeadEndTM Fluorometric kit (Promega Corp, Madison, WI) according to the manufacturer's protocol. Briefly, after incubation with equilibration buffer, the specimens were incubated in a solution of Nucleotide Mix and rTdT enzyme in equilibration buffer at 37°C; and then unincorporated fluorescein-12-dUTP was removed by washing with PBS. The number of green fluorescence-labeled cells in the specimen was then determined.

### Statistical analysis

Data were presented as mean ± standard deviation (SD). Two-side paired or non-paired student's t-test was used to compare the mean values of two sets of data. ANOVA tests were used to analyze the mean values of multiple group data. Differences were considered significant at *p*<0.05.
